# High-Precision Contactless Stereo Acoustic Monitoring in Polysomnographic Studies of Children

**DOI:** 10.3390/s25165093

**Published:** 2025-08-16

**Authors:** Milan Smetana, Ladislav Janousek

**Affiliations:** Department of Electromagnetic and Biomedical Engineering, Faculty of Electrical Engineering and Information Technology, University of Zilina, Univerzitna 8215/1, 010 26 Zilina, Slovakia; ladislav.janousek@uniza.sk

**Keywords:** sleep sounds, stereophony, neural networks, deep learning, classification of sleep sounds, polysomnography

## Abstract

**Highlights:**

**What are the main findings?**

- Robust and cost-effective measurement set-up: The system is designed to ensure re-liability in sound analysis while being cost-effective for long-term use.
- Crucial for doctors: A critical knowledge base is essential for healthcare professionals to make informed decisions and implement effective therapies for pediatric patients.

**What is the implication of the main finding?**

- High accuracy of achieved results: Using time-domain analysis, we demonstrated a detailed representation of sound architecture. The results ensure reliability in the categorization process. The proposed LSTM neural network, trained on a dataset of 1500 sounds per category, features two layers. The deep neural network achieved 91.16% overall, with individual channel accuracy reaching 93.35%.

**Abstract:**

This paper focuses on designing a robust stereophonic measurement set-up for sound sleep recording. The system is employed throughout the night during polysomnographic examinations of children in a pediatric sleep laboratory at a university hospital. Deep learning methods were used to classify the sounds in the recordings into four categories (snoring, breathing, silence, and other sounds). Specifically, a recurrent neural network with two long short-term memory layers was employed for classification. The network was trained using a dataset containing 1500 sounds from each category. The deep neural network achieved an accuracy of 91.16%. We developed an innovative algorithm for sound classification, which was optimized for accuracy. The results were presented in a detailed report, which included graphical representations and sound categorization throughout the night.

## 1. Introduction

Sleep is one of the primary physiological states of the human body. It is crucial for the proper functioning of the autonomic nervous system, as well as cognitive and emotional health. Insufficient and poor-quality sleep is associated with excessive daytime sleepiness, neurocognitive impairment, and increased risk of accidents and cardiovascular diseases. More than a billion people worldwide suffer from sleep deprivation. Most people are unaware of their sleep disorders, which poses a significant challenge for healthcare systems and has a profound impact on public health. Epidemiological studies currently show that more than a third of the population suffers from sleep disorders.

As a result, sleep monitoring technologies have become essential in modern medical diagnostics. To streamline the expert analysis process and reduce subjectivity, algorithms have been developed in recent decades to automatically determine sleep phases based on EEG analysis. In a study [1], a deep learning network called DeepSleepNet was proposed for the automatic grading of sleep stages based on raw single-channel EEG data. Most research utilizes hand-engineered features, which require prior knowledge of sleep analysis techniques. Only a few studies have incorporated temporal information, such as transition rules, into encoding. Their innovative model uses bidirectional long short-term memory to learn transition rules between sleep stages from EEG epochs, using convolutional neural networks to extract time-invariant features. The results showed that our model’s overall accuracy and macro F1-score were comparable to those of the most advanced techniques on both datasets (MASS: 86.2% vs. 81.7%, Sleep-EDF: 82.0% vs. 76.9%; MASS: 85.9% vs. 80.5%, Sleep-EDF: 78.9% vs. 73.7%).

Contact sensors, commonly used in sleep research, can affect results and disrupt standard sleep patterns. Additionally, there are limited sleep studies available, leading to many individuals with sleep disorders going undiagnosed. Study [2] introduces a new method for estimating wake staging, rapid eye movement (REM), and non-REM sleep using sleep sound analysis. The researchers trained one-layer feedforward neural network classifiers using audio recordings from 250 patients undergoing polysomnography (PSG) research at a sleep laboratory which employed non-contact microphones. Following validation, the audio-based system demonstrated an 87% agreement with PSG in 30 s segments, with a Cohen’s kappa of 0.7.

Several studies have addressed the issue of detecting snoring sounds from recorded snoring sounds during sleep using signal processing algorithms and pattern recognition. Snoring is a common disorder with negative social and health impacts. In paper [3], a technique is described which allows for the automatic monitoring of snoring features, such as frequency and severity, using audio data from a standalone microphone. This technique provides an automatic and portable alternative to polysomnography. The system uses spectral characteristics and Hidden Markov models (HMMs) to model different types of sounds. To test the system, snoring noises in new audio recordings were automatically classified and compared to human annotations. Despite a limited training set, the method detects and analyzes snoring sounds, achieving 82–89% accuracy.

Cavusoglu et al. [4] presented a method for identifying snoring episodes in sleep sound recordings. They used the energy distributions of sub-bands in sleep sound segments, also known as “sound episodes,” to classify sleepers as either snores or non-snores. They employed principal component analysis to categorize episodes in two dimensions effectively and to distinguish between snores and non-snores. The system was tested using handwritten remarks from an ENT specialist as a guide. The algorithm achieved 97.3% accuracy for basic snorers. However, when the training data included both obstructive sleep apnea (OSA) patients and simple snorers, the accuracy decreased to 90.2%. For detecting snore episodes in OSA patients, the accuracy rate was 86.8%.

Levartovsky et al. conducted a study on snoring using a sound level metre technique [5]. Their research found that men tend to snore more loudly, which is associated with a higher apnea–hypopnea index (AHI). They developed an algorithm to detect and analyze breathing and snoring sounds and systematically investigated how factors such as gender, sleep stages, and AHI affected the characteristics of snoring. The study examined over 290,000 instances of breathing and snoring (above 50 dB). The results showed that men had a 23% higher snoring index (events/h, SI) compared to women (*p* = 0.04). Both men’s and women’s snoring index decreased by 50% throughout their sleep (*p* < 0.01). This technique allows for the systematic identification and examination of snoring and breathing noises from an entire nighttime recording. Research has shown that over 97% of snoring occurs during inspiration. During expiration, these sound events are not expected [5]. Research has also examined methods to determine and classify “snoring” and “non-snoring” events throughout the night.

Algorithms have been developed using a non-contact microphone to detect snoring events throughout the night. The goal of the study [6] was to create and validate a reliable, high-performance, and sensitive snore detector that operates throughout the entire night using non-contact technology. During PSG, sounds were captured with a directional condenser microphone positioned one metre above the bed. An AdaBoost classifier was trained and validated using manually labelled snoring and non-snoring acoustic events. To train the sound detector, over 76,600 acoustic episodes collected during the study’s design phase were manually classified into snore and non-snore episodes by three scorers. Features extracted from the time and spectral domains of the audio enable accurate distinction between snoring and non-snoring events. This method allows for the detection and analysis of snoring sounds throughout the entire night, providing quantifiable measures for the objective monitoring of patients. Through a ten-fold cross-validation technique, the average snore/non-snore detection rate (accuracy) for the design group was 98.4%. When tested on the validation group, the average detection rate was 98.2%, with a sensitivity of 98.0% (identifying snoring as snoring) and a specificity of 98.3% (identifying noise as noise).

Additionally, many studies categorize “snorers” into two groups, pathological and non-pathological, and distinguish between natural snoring and snoring that is either simulated or induced by sleep medication [7]. Studies on the relationship between OSA and snoring have shown that they receive the most extensive coverage of other sounds occurring during sleep [8]. Moreover, validated procedures that, based on sophisticated acoustic analysis, can discriminate subjects according to the AHI. The findings from this study support the hypothesis that OSA is associated with functional abnormalities of the upper airway; several studies related to this topic address snoring and its detection [9]. Individual microphones were also examined in terms of their usefulness in recording night sounds with relatively low intensity [10]. Several studies have shown that it is possible to detect breathing sounds accurately and to discriminate between sleep and wakefulness using audio analysis [11,12,13]. The findings regarding differences between REM and NREM snoring have also been identified [14,15]. One of the groundbreaking studies is the work that distinguished between macrophagic sleep (REM, NREM, wake) based on all-night sound recordings and subsequent online and offline analysis [16,17,18,19]. All procedures are compared with PSG data. Table 1 briefly summarizes the studies conducted that deal with sleep classification.

This paper focuses on designing, implementing, and realizing an advanced measurement set-up for recording sounds during the sleep of children undergoing polysomnographic examinations in a specialized medical laboratory. The paper provides a detailed analysis of the entire process, including the obtained results. A proposed LSTM deep neural network with two architectural layers is utilized to ensure high accuracy. This procedure aims to develop a reliable and cost-effective measurement system for analyzing sound patterns through graphical categorization. The measurement output is a clear and concise report that provides valuable information for healthcare professionals. This knowledge base can then be utilized to administer more effective therapy in an environment that prioritizes patient well-being.

The main innovations of our approach are the use of stereo acoustics in polysomnography of children, contact-independent signal collection compatible with the clinical environment, implementation of an effective and fast classification chain, and comparison with traditional polysomnographic signals (EEG, EMG, EOG).

## 2. Proposed Methods and Algorithms

It is essential to note that there are currently no established, valid standards for recording sleep sounds using non-contact methods, and procedures exhibit significant variability from study to study. For this reason, the following will also be presented with fundamental aspects and recommendations. Stereophonic capture is used to record sounds for sleep, but sound recording has been minimal so far, and its aspects have yet to be investigated. The proposed AB (Microphone A, Microphone B) system uses two spatially separated microphones. This configuration is called time-division or differentiated stereo. If circular microphones are used, it is classified as time-domain stereo. If carotid microphones are used, the system becomes an intensity-time stereo system.

**Figure 1:**
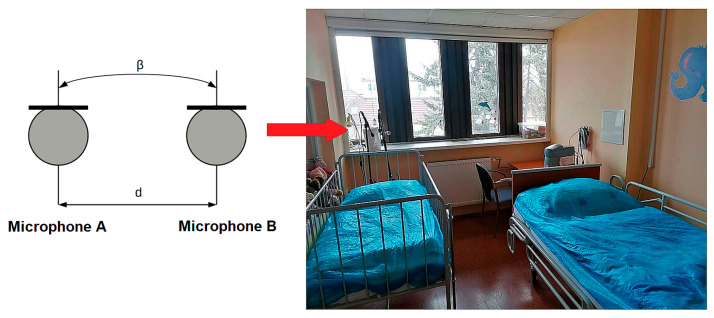
A schematic representation of the AB sensing system and its practical implementation at the laboratory (Children’s Sleep Laboratory of the Clinic for Children and Adolescents, Centre for Rare Sleep and Wake Disorders in Childhood, University Hospital Martin, Slovak Republic).

The microphones shall be placed at a distance from each other. This distance is called the base. In Figure 1, the letter “d” denotes the base. If the width of the base is 40–80 cm, it is a small AB, and if the width of the base is larger, it is a large AB. Theoretically, this value can be up to several metres. It specified a recommended base width of 40–60 cm, based on the consideration that human hearing has a reduced ability to localize low frequencies (below 150 Hz) by up to ¼. Theoretically, the sensitivity of microphones with circular characteristics should be equal for all directions; practically, this is not the case. In the high-frequency range, these microphones exhibit some degree of directionality. Therefore, a rotation angle β ranges from 0° to 90°. In an AB system, the mid-range of the reproducing base is present. In extreme cases, a complete rupture of the stereo image may occur, or a ping-pong effect may occur.

When working with sound recordings that have been captured all night, it is not easy to identify and classify sounds accurately without signal processing. Various methods are employed to enhance the signal-to-noise ratio (SNR). Speech enhancement algorithms, also known as noise cancellation algorithms, help to reduce or eliminate background noise. These algorithms are typically used to enhance speech quality and are categorized into three main groups: spectral analysis methods, methods based on statistical models, and methods based on subspace algorithms.

**Figure 2:**
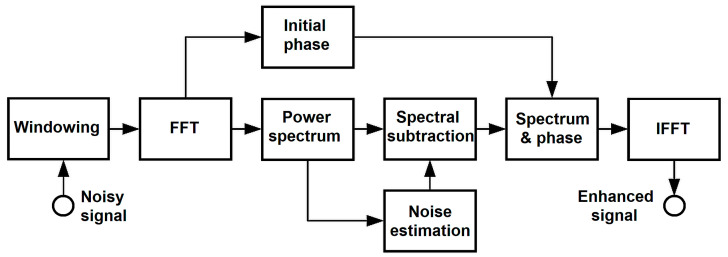
Block diagram of the algorithm used in the spectral subtraction method.

Spectral reading methods are also helpful in reducing noise in acoustic signals obtained in sleep laboratories. This method is suitable for recovering the power or amplitude spectrum of a signal that is observed in a noisy environment. The basic principle of this method assumes that it is possible to estimate the spectrum of a pure signal by subtracting the spectrum of the estimated noise from the spectrum of the original noisy signal, assuming that the noise is additive, as shown in Figure 2.

The noise spectrum can be estimated from areas where no valid signal is present. It is essential to note that this method assumes the noise is stationary or changes only slowly, and therefore, the noise spectrum remains relatively constant. The enhanced signal is obtained by performing an inverse Fourier transform of the estimated signal spectrum using the phase of the signal with noise. The advantage of this algorithm is that it is computationally undemanding, requiring only direct and inverse Fourier transforms. However, it is essential to apply this method carefully to prevent significant loss of helpful information and signal distortion. In spectral analysis, the input signal *y*(*m*) is divided into frames consisting of *N* samples each. Every frame is multiplied by a window function (e.g., Hanning, Hamming) and then transformed using the Discrete Fourier Transform (DFT) into *N* spectral samples. The purpose of using the window function is to reduce the impact of signal discontinuities at the frame boundaries. The modified signal can be represented as follows:

(1) $$y_{w}\left(m\right)=w\left(m\right)y(m)$$

(2) $$y_{w}\left(m\right)=w\left(m\right)\left[x\left(m\right)+n\left(m\right)\right]$$

(3) $$y_{w}\left(m\right)=x_{w}\left(m\right)+n_{w}\left(m\right)$$

where *y*_w_(*m*) is the weighted input signal, *w*(*m*) is the weighting window, *x*_w_(*m*) is the weighted clean signal, and *n*_w_(*m*) is the weighted noise signal. The weighting operation can be expressed in the frequency domain:

(4) $$Y_{w}\left(k\right)=W\left(k\right)*Y(k)$$

(5) $$Y_{w}\left(k\right)=X_{W}\left(k\right)+N_{W}\left(k\right)$$

where *Y*_w_(*k*) is the frequency spectrum of the weighted signal, *W*(*k*) is the frequency spectrum of the dedicated window, *X*_w_(*k*) is the frequency spectrum of the pure weighted signal, and *N*_w_(*k*) is the frequency spectrum of the weighted noise signal. The operator ∗ represents the convolution operation. All signals are weighted, and therefore, the subscript *w* will not be mentioned further.

The spectral subtraction equation is

(6) $${\hat{X}}_{k}^{b}=Y_{k}^{b}-\alpha \left(k\right)\bar{N_{k}^{b}}\;.$$

Here, ${\hat{X}}_{k}^{b}$ is the estimate of the amplitude spectrum of the pure signal to the power of *b*, $Y_{k}^{b}$ is the spectrum of the input signal, and $\bar{N_{k}^{b}}$ is the average amplitude of the noise spectrum multiplied by *b*. The noise is assumed to be stationary and random. For amplitude spectral reading, the exponent *b* = 1 and for power spectral readout, *b* = 2. The coefficient *α*(*k*) controls the amount of noise to be subtracted from the noisy input signal. For complete noise subtraction, *α*(*k*) = 1. If *α*(*k*) > 1, then excessive noise subtraction occurs. The average noise spectrum is obtained from the original signal in sections where useful information is not present and can be expressed as

(7) $$\bar{N_{k}^{b}}=\frac{1}{M}\sum_{i=0}^{M-1}N_{k,i}^{b}\;,$$

where $N_{k,i}$ represents the spectrum of the *i*-th noise frame at discrete frequency *k*, assuming there are only *M* frames.

The enhanced amplitude spectrum estimate is combined with the phase of the noise signal and then converted to the time domain using the Inverse Fast Fourier Transform (IFFT).

(8) $${\hat{x}}_{k}=\sum_{k=0}^{N-1}\left({\hat{X}}_{k}e^{j\theta_{Y_{k}}\;}\right)e^{j\frac{2\pi }{N}km},\;\;\;\;\;\;\;\;\;\;\;\;\;\;\;m=0,\ldots ,\;N-1\;\;\;\;\;$$

where ${\hat{x}}_{k}$ is the signal in the time domain after IFFT, ${\hat{X}}_{k}$ is the estimate of the amplitude spectrum of the improved signal, and $\theta_{Y_{k}}$ is the phase of the noisy signal $Y_{k}$ at the corresponding frequency *k*. The spectral subtraction method capitalizes on the property of the human auditory system, which does not perceive a phase change. Audible noise is mainly caused by the distortion of the amplitude spectrum, and the method does not consider the amplitude spectrum in the process [16]

Recurrent Neural Networks (RNNs) are a type of Artificial Neural Networks (ANNs) that excel at learning sequences of data and are categorized as Feedforward Neural Networks. They are well-suited for tasks involving sequential data as they can consider both current and past input. When working with input sequences of varying lengths and where individual items impact the overall output, RNNs can incorporate an internal state within the network, typically achieved through a feedback connection [17,18,19].

The process of transferring information from the previous iteration to the hidden layer can be mathematically described. The representation of the hidden state and input at a specific time *t* step is **H***_t_* ∈ ℝ*^n^*^×*h*^ and **X***_t_* ∈ ℝ*^n^*^×*d*^, where *n* is the number of samples, *d* is the number of inputs for each sample, and *h* is the number of hidden units. **W***_xh_* ∈ ℝ*^n^*^×*h*^ is the weight matrix, **W***_hh_* ∈ ℝ*^h^*^×*h*^ represents the hidden-state-to-hidden-state matrix, and **b***_h_* ∈ ℝ^1×*h*^ is the bias parameter. All this information is then passed through an activation function *ϕ* (e.g., sigmoidal), which adjusts the gradients for use in backpropagation. The equation for the hidden variable can be obtained based on the following definitions:

(9) $$H_{t}=\phi_{h}\left(X_{t}W_{xh}+H_{t-1}W_{hh}+\;b_{h}\right).$$

The output variable **O***_t_* is determined by the following expression:

(10) $$O_{t}=\phi_{o}\left(H_{t}W_{h0}+\;b_{0}\right).$$

At time *t*, the hidden state (**H***_t_*) includes the previous hidden state at time *t* − 1 (**H**_(*t*−1)_) for each time step. This allows the RNN to store information about all previous hidden states leading up to **H**_(*t*−1)_, including **H**_(*t*−1)_ itself.

RNNs utilize the Backpropagation Through Time (BPTT) algorithm for error backpropagation. In this algorithm, the gradient from the previous layer is consistently multiplied by the weight. This can lead to issues associated with exploding gradients (value greater than 1) and vanishing gradients (falling to 0), resulting in incorrect algorithm operation. Other types of RNNs, such as Echo State Networks (ESN) or Long Short-Term Memory (LSTM) networks, have been developed to address and resolve this problem [20,21,22,23,24].

## 3. Experimental Set-Up

**Figure 3:**
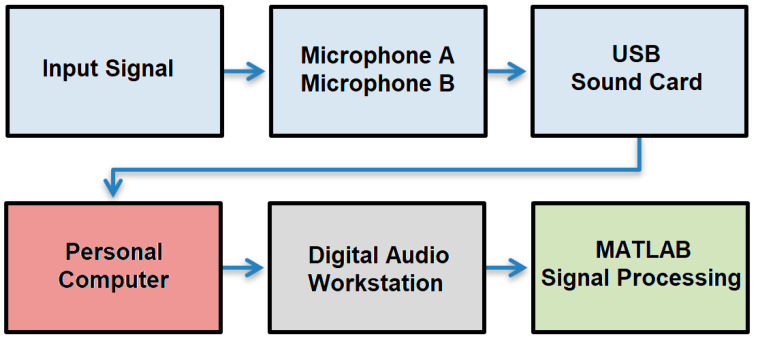
Block diagram of the designed measuring assembly.

System design and verification: Stereophonic sound recording techniques provide higher-quality reproduction by using two audio tracks (L and R channels). This is important for sleep sounds as it ensures that helpful information is not lost if the patient changes position. To achieve high-quality recordings with minimal background noise, it is crucial to utilize devices with suitable electroacoustic properties. The recording process uses a pair of microphones mounted on a microphone stand with gallows. The signal is transmitted through shielded cables with XLR connectors to an external USB sound card. This sound card is then connected to the computer via a USB-C to USB interface. The sound is captured on the computer using DAW software version 4.6.2 (Digital Audio Workstation), Figure 3.

We selected a pair of Superlux ECM999 microphones (Superlux Enterprise Development Ltd., Shanghai, China) for recording. These are condenser microphones designed for use in testing, measuring, and recording applications. The manufacturer claims that the microphone has several room analysis systems. The microphone provides accurate sound response and covers the whole audible spectrum (20 Hz–20 kHz). This type of microphone is also used for recording vocal–instrumental events or capturing the general atmosphere of rooms. It requires phantom power within the range of 9 V to 52 V for power supply and has a maximum volume level of 128 dB. The detailed technical specifications of the microphone are as follows: microphone type: condenser, electret, Ø 0.24 “1/4” pre-polarized condenser capsule; directional characteristic: omnidirectional; frequency response: 20 Hz–20,000 Hz; sensitivity: −37 dBV/Pa (14 mV); output/min. load impedance: 200 Ω/1 kΩ; SNR: 70 dB; maximum sound pressure level: 132 dB; dynamic range: 10 dB; power supply: phantom power, 12–52 V DC, 2 mA; polar pattern: omnidirectional; and equivalent output noise: 24 dB. Regarding the various adjustment options, we used microphone stands with Lewitt TMS131 booms (Lewitt GmbH, Vienna, Austria). For accurate simultaneous recording using two microphones, we used a universal USB audio converter, the Focusrite Scarlett 2i2 3rd generation (Focusrite Audio Engineering Ltd., Hertfordshire, UK). This external sound card has two combined inputs, two-line outputs, and a headphone output (6.3 mm jack) with separate volume control. It features high-quality A/D and D/A converters with 24-bit/192 kHz resolution, as well as microphone preamplifiers. The sound card offers low distortion, low latency, and a high SNR of more than 100 dB.

Before starting the recording, it is necessary to set the gain and other parameters correctly. For this purpose, closed studio headphones, the Superlux HD662 EVO (Superlux Enterprise Development, Lianyungang, China), were used. These headphones reproduce sound with a wide frequency response, ranging from 10 Hz to 30 kHz, at a nominal impedance of 32 Ω, and have a maximum output power of 200 mW.

An essential component in this measurement set-up is the use of an appropriate computer. When conducting overnight recordings, it is crucial to pay close attention to this aspect, as recording audio at a sampling frequency of 48 kHz results in large data files. After exporting the signal to Waveform Audio File (WAV) format, a 5- to 7 h recording represents approximately 8 to 12 GB of data. To ensure seamless recording, processing, and analysis of this volume of data, it is advisable to use a more powerful computing platform. Taking these factors into account, a desktop PC was assembled to satisfy the requirements of this task.

We conducted a series of all-night test recordings using the assembled measuring system. The initial measurements were used to calibrate the analogue gain at the sound card’s input and to determine the microphone position (base and rotation β). We found that an input gain of 50–75% (corresponding to 28 dB–42 dB) worked well. We tested the professional DAW software Pro Tools First and Audacity for recording sound to a PC. Pro Tools First was not suitable due to its complex user interface and the lengthy export process required to convert to WAV format. As a result, we opted for simpler Audacity software version 2.4. When digitizing sound accurately, it is essential to adhere to the sampling theorem. For audible sound in the 16 Hz–20 kHz range, the sampling frequency must be at least 40 kHz. In practice, a 10% increase is often used, resulting in a sampling frequency of 44.1 kHz. During our testing, we used sampling frequencies of 44.1 kHz, 44.8 kHz, and 96 kHz. We found that a sampling frequency of 44.8 kHz was optimal for our purposes.

**Figure 4:**
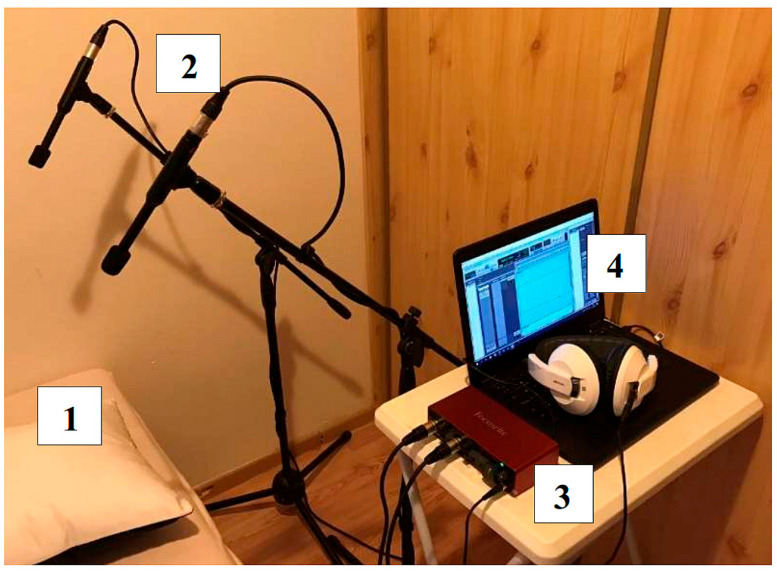
Measurement set-up: improvised laboratory for preliminary testing of the apparatus: 1: bed for proband, 2: pair of two condenser microphones, 3: USB sound card/AD converter, 4: computer and headphones.

Implementation of the designed system in the pediatric sleep laboratory: The measuring apparatus was first tested in an improvised laboratory (Figure 4). After verifying the correctness of the entire system and its functionalities, it was then placed in a specialized laboratory. Subsequently, the assembly was then implemented in the Children’s Sleep Laboratory at the University Hospital Martin (UHM, Slovak Republic), Clinic for Children and Adolescents. We conducted six all-night sound recordings during sleep simultaneously with the PSG examination. The recordings lasted 5–8 h each, with a sampling frequency of 44.8 kHz and a 24-bit resolution. The microphones were positioned 45 cm above the patient’s head to capture the sleep sounds with the highest possible intensity.

Additionally, a video-polysomnographic examination was performed in this sleep laboratory, allowing us to link image and sound and compare the recordings obtained using a piezoelectric microphone placed in the patient’s jugular fossa with those from a pair of non-contact microphones. We used the Alice 6 LDxS (Koninklijke Philips N.V., Amsterdam, The Netherlands) polysomnograph to obtain PSG recordings, and the data were recorded and scored using the Sleepware G3 software version 3.9.0 (Philips Respironics, Amsterdam, The Netherlands). The PSG examination resulted in a detailed report. To synchronize the audio recording with the PSG recording, a night vision camera was employed. Subjects and their parents were informed of the ongoing recording and provided with consent. All legal and ethical considerations regarding the subjects were observed in accordance with the applicable GDPR and the statements of the Ethics Committee and the Regional Public Health Authority. Table 2 provides basic information about the subjects and the records obtained. The ages of the patients ranged from 5 to 14 years, with recordings obtained from two female subjects and four male subjects.

**Figure 5:**
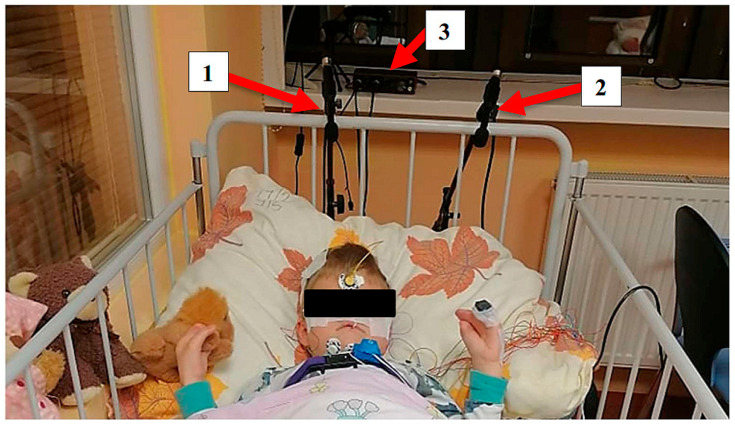
Measuring assembly: spatial configuration during the experiments’ realization, Children’s Sleep Laboratory, University Hospital Martin, Slovak Republic; 1: condenser microphone A, 2: condenser microphone B, 3: USB sound card/AD converter.

Figure 5 illustrates the placement of the measurement equipment in the sleep laboratory environment during the ongoing PSG examination. In certain instances, it can be challenging to obtain reliable recordings due to the restlessness and discomfort experienced by young patients caused by the numerous electrodes attached to their bodies. Additionally, the presence of a parent can also negatively impact the recording quality, as their actions, such as loud snoring and movements, can introduce significant interference.

## 4. Processing of Measured Signals, Training, and Validation

**Figure 6:**
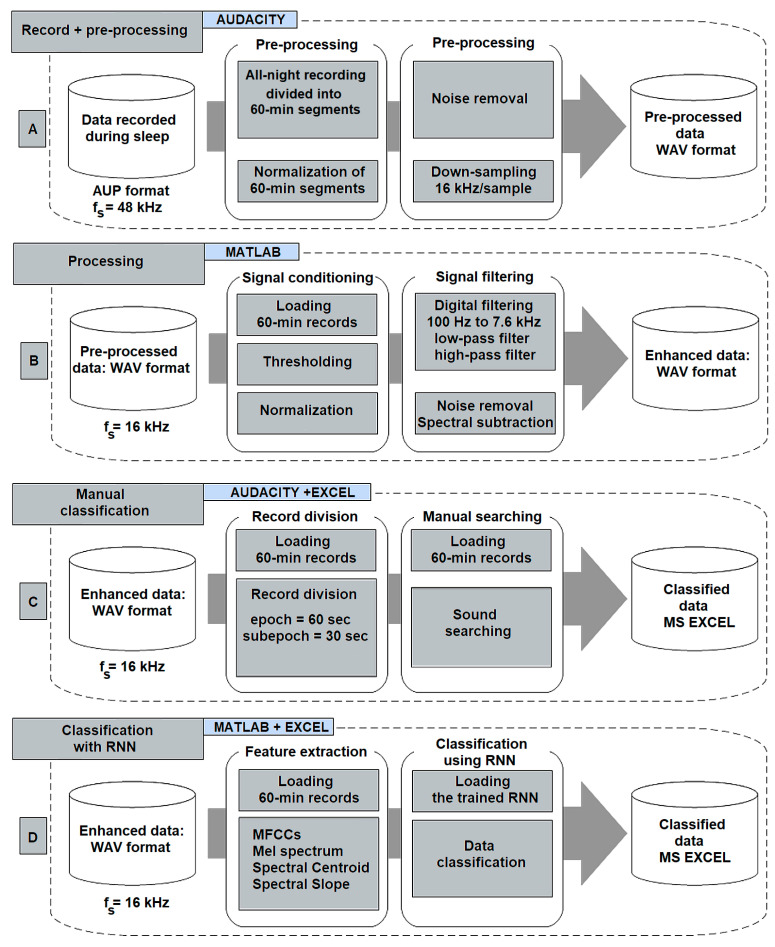
Overview diagram of the proposed method of the audio recordings: (**A**) record and signal pre-processing; (**B**) signal processing; (**C**) manual classification; (**D**) classification using RNN.

In the previous section, we discussed the set-up for taking measurements and recording all-night sessions from six subjects. The recordings were initially saved in AUP format (audio project file created by Audacity) and later converted to WAV format. The processed records are analyzed for results. An overview of the approach used in the pre-processing, processing, and classification of sound data is presented in Figure 6 and discussed below. This figure provides a comprehensive schematic overview of the proposed method for pre-processing, processing, and classification of audio recordings acquired during polysomnographic studies. The diagram outlines the sequential pipeline, beginning with the input of raw audio signals, which are subjected to noise reduction and segmentation in the pre-processing stage. These segments are then transformed into time–frequency representations, which serve as input to a CNN for spatial feature extraction. Subsequently, dependencies are captured using an LSTM layer, and the extracted high-level features are passed to a fully connected classification layer. The final output consists of class probabilities corresponding to specific acoustic events relevant to sleep diagnostics.

Signal pre-processing: Following calibration and test measurements, acquisition was performed, consisting of six all-night recordings with a sampling frequency of 44.8 kHz and a bit resolution of 24 bits, stored in AUP format. The signal is pre-processed in the following steps: the 5–8 h overnight recording is shortened to 5 h. It is then divided into 60 min sections, synchronized with the video-polysomnographic recording. Each section is normalized to a range of −1 to 1 and a level of 0.0 dB to increase the sound signal level without loss of quality. The noise reduction function is applied to partially eliminate the noise in the modified signal at this stage. The modified audio signal is then subsampled to a sampling frequency of 16 kHz for faster processing in MATLAB R2020a (MathWorks, Inc., Natick, MA, USA) and exported to WAV format. The result was pre-processed data in WAV format, ready for further processing. These steps are performed independently for both channels.

Signal processing: The pre-processed signal was further analyzed using advanced algorithms for feature extraction and classification using MATLAB. An extensive script was developed, incorporating multiple functions, which we will explore in detail. The signal processing is carried out in the following steps.

**Figure 7:**
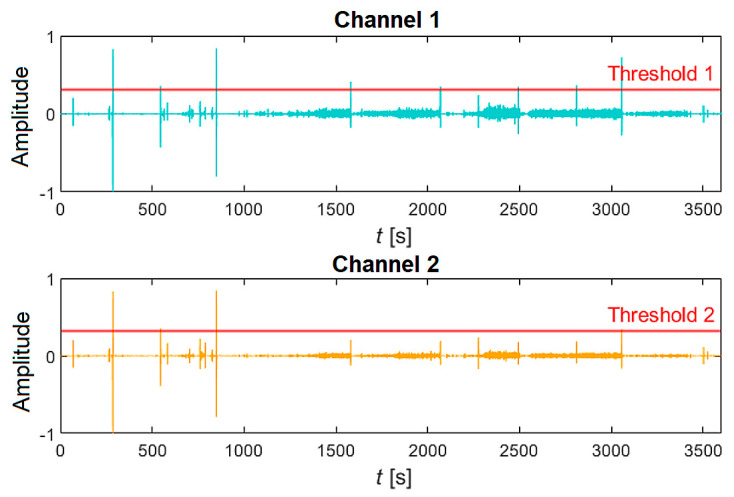
Removing unwanted peaks from a 60 min signal. Interactively selected threshold levels (Threshold 1 and Threshold 2).

**Figure 8:**
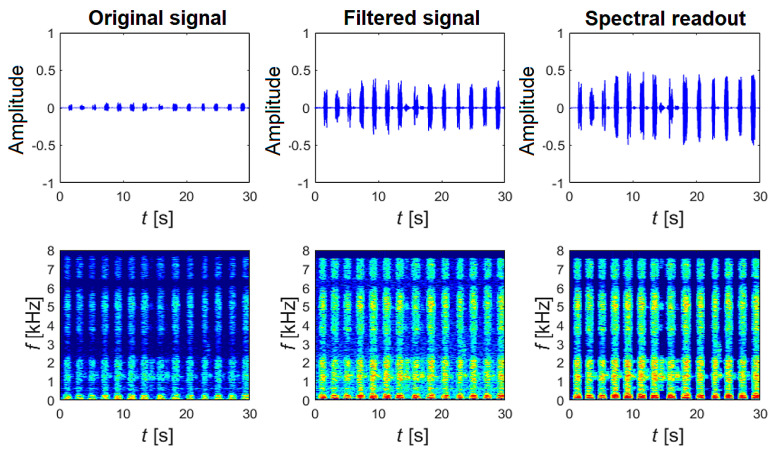
Selected 30 s sub-epoch of the entire overnight recording, showing the signal’s time course and spectrograms for the original signal, the filtered signal, and after applying multi-band spectral subtraction on the 1st channel.

The first step involves loading the hourly recording (both channels) with a sampling frequency of 16 kHz into the InputData structure using the LoadAudioData function. If the recording exceeds 60 min, it is automatically shortened to the required number of samples (57.6 × 10^6^ samples for both channels). Sleep sound signals commonly contain high peaks (e.g., door slamming) that need to be eliminated. The PeaksExtraction function, which has been designed and created, enables the interactive selection of a specific threshold value above which the signals will be clipped, Figure 7. If there are no peaks in the signal, this step can be skipped. Then, the modified signal undergoes filtering using a pair of digital finite impulse response (FIR) filters. The first filter is a low-pass filter with a cut-off frequency of 7.5 kHz, followed by a high-pass filter with a cut-off frequency of 100 Hz. The filter delay was determined using the “grpdelay” function, and the corresponding delay samples were removed from the records. Afterwards, a method called spectral subtraction is applied to suppress noise and improve the signal within the desired frequency band (100 Hz–7.5 kHz). Specifically, a multiband spectral subtraction algorithm, commonly used for enhancing speech signals, was utilized. Finally, the processing is visually inspected for accuracy, and relevant oscillograms and spectrograms are generated, as shown in Figure 8.

**Figure 9:**
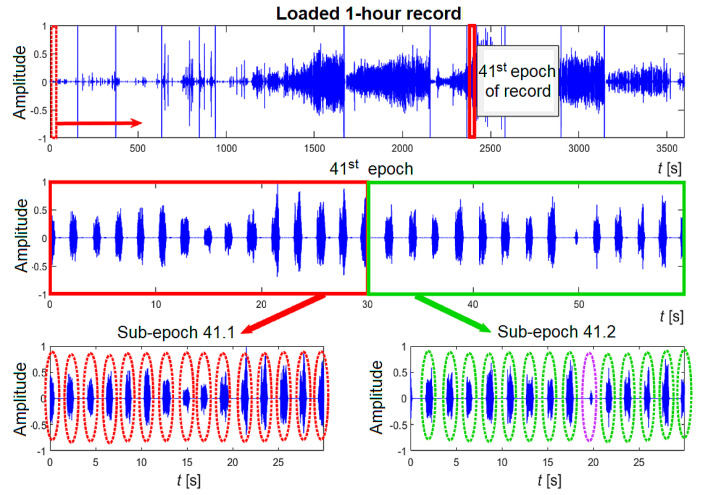
Schematically shown division of the signal into epochs and sub-epochs, the 41st epoch of record No. 3 (channel 1) is displayed.

Manual classification: To obtain and interpret information from the processed signal, it is necessary to evaluate sound events related to different sleep patterns manually. The sounds are classified into four categories: 1: snoring, 2: breathing, 3: silence, and 4: other sounds (movements, speech, noise). The manual scoring method proposed in this work involves the following steps. The overnight recording (5 h) undergoes pre-processing and is then loaded into Audacity. The 1st hour is loaded first, followed by the 2nd hour, and so on. Each hour is divided into 60 sections of equal length called 60 s epochs. Each epoch is further divided into two 30 s sub-epochs, which are correlated with the epochs of the video-polysomnographic recording. Figure 9 illustrates the manual evaluation process for each epoch (60 s) or each sub-epoch (30 s) of the recording.

**Figure 10:**
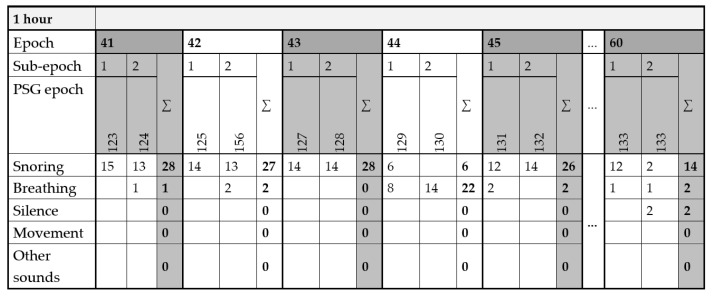
Recording of events in individual epochs during manual scoring.

During segmented periods, events are categorized and recorded. For instance, in Figure 9, during sub-epoch 41.1, 15 snoring events (indicated by the red ellipse) were identified. In sub-epoch 41.2, there were 13 snoring events (green ellipses) and one breathing event (purple ellipse). The recorded events in each sub-epoch are totalled and entered into a prepared EXCEL form. This form is divided into the appropriate number of hours, epochs, sub-epochs, and five categories. An example of the form for recording events in individual epochs is presented in Figure 10. The table also combines the category movements with other sounds. It is noteworthy that only episodes lasting over 5 s are considered as silence. Therefore, a brief interval of silence between two snoring sounds is not classified as silence to avoid numerous redundant silence events. The results of the manual classification consist of 5 forms, with manually added events designated for specific categories. Manual classification is crucial for creating a dataset for individual categories and preparing for classification using RNNs.

Recurrent Neural Network Classification: The approach utilizes an RNN for classification. The LSTM network with two layers was chosen for classification. Before creating the neural network, a dataset containing sounds of snoring, breathing, silence, and other sounds was prepared. Each sound in the dataset has a length of 1 s. There are 1500 sounds in each category, making a total of 6000 sounds in the dataset, which is considered relatively small. It has been demonstrated that RNN-based classifiers can achieve high accuracy even with smaller training datasets. During the preparatory phase, attempts were made to train the LSTM network with smaller numbers of sounds in each category (500, 1000). However, the accuracy with such a small amount of training data ranged from approximately 75% to 85%, which was not deemed sufficient. Therefore, the dataset size was increased. The exact division of the dataset into training and validation data is shown in Table 3.

The neural network was developed manually. Commands were used to define its architecture and parameters. The construction of the network involved defining the individual layers of the model. We used a neural network structure consisting of 8 layers: the Sequence Input Layer, which serves as an input layer for sequence data, using a 47-dimensional sequential input; the LSTM layer, lstmLayer_1, learns long-term dependencies between time steps in sequential data, containing 125 hidden units; the dropout layer, dropout_1: during training, it randomly sets a certain number of input elements to zero with a certain probability and thus prevents overtraining of the network (for the input layer, the dropout value was set to 0.2); the LSTM layer, lstmLayer_2: the second LSTM layer consists of 150 hidden units; the dropout layer, dropout_2: the dropout value for this layer is also 0.2; the fully connected layer, fc: connects every neuron in one layer with all neurons in the next layer, multiplying the input by a weight matrix and adding a bias vector; the Softmax layer: this layer applies the normalization of outputs; and the classification layer: this layer calculates cross-entropy loss for classification and mutual classification of mutually exclusive classes (snoring, breathing, silence, other).

**Figure 11:**
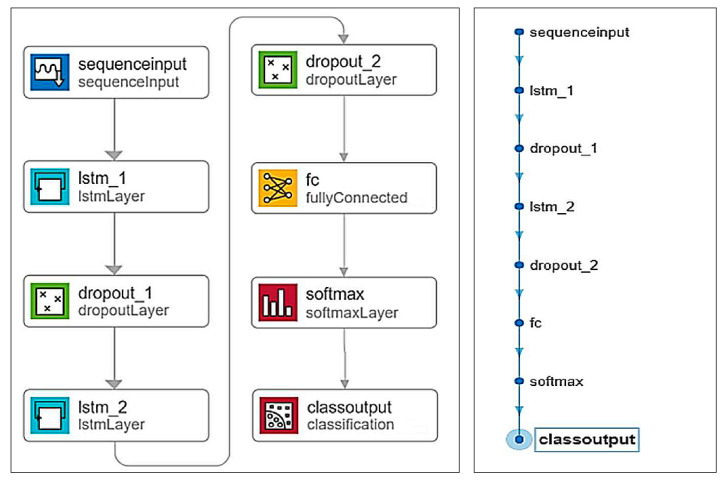
Architecture of the used LSTM network.

The LSTM network with two layers was chosen for classification, which is deemed sufficient even for tackling more complex problems. The network architecture is depicted schematically in Figure 11.

Feature extraction from training and validation data precedes the training of the neural network. Audio data contains a large amount of redundant information. This redundant information (high dimensionality) can be reduced by first extracting certain features and then training the model using the extracted features. In MATLAB, the Audio Feature Extractor function is used for this. In the proposed model, four features are extracted: MFCC, Mel-spectrum, spectral centre (Spectral Centroid), and spectral slope (Spectral Slope).

**Figure 12:**
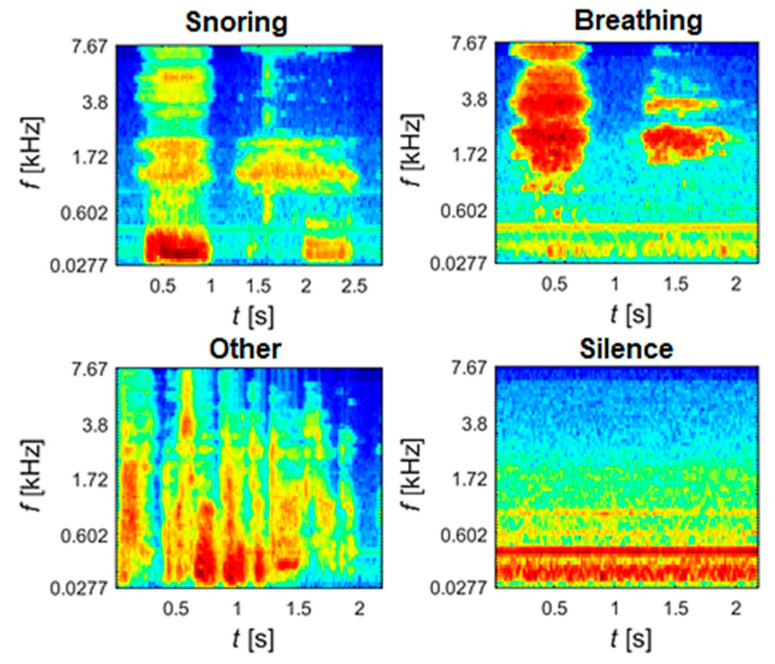
Mel-spectrograms for 4 classes (different categories).

MFCC and Mel-spectrum are commonly used to represent audio signals. MFCC extraction has produced good results in snoring recognition. When calculating Mel-spectrograms, the signal is first pre-processed in the time domain through framing and windowing. Then, the FFT is calculated for each frame to obtain the power spectrum. Mel-filters are applied to power spectra to convert linear frequencies to logarithmic ones, resulting in a Mel-spectrogram, Figure 12.

From the Mel-spectra, the spectral centroid is calculated. It is a feature of the spectrum used which shows the centre of gravity of the spectrum, in this case, the Mel-spectra. On the other hand, spectral slope in digital signal processing shows how quickly the audio signal’s spectrum moves towards high frequencies, calculated using linear regression.

**Figure 13:**
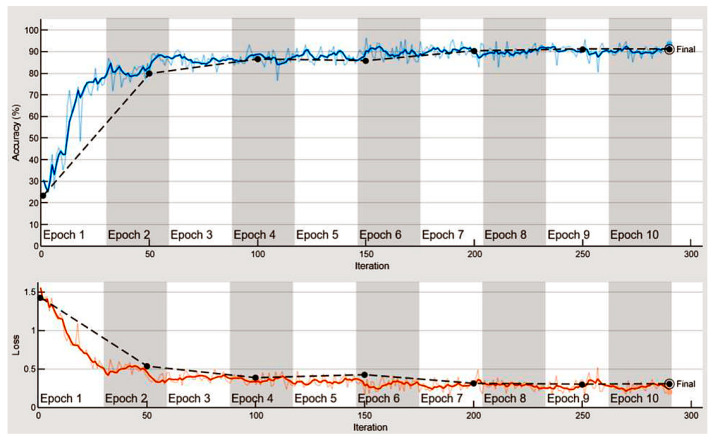
LSTM network training progress (10 epochs) achieved accuracy: >91%.

To create a neural network model, the final step is to train the LSTM network and configure its parameters. The training process consisted of 10 epochs, with a mini-batch size of 128 and a learning rate of 1 × 10^−3^. Training the network took approximately 35 min using an AMD Ryzen 5 3600X CPU (Santa Clara, CA, USA). The training progress can be monitored to check the training and validation accuracy immediately, as shown in Figure 13. With a larger training dataset, the learning process would take several hours, depending on the hardware and parameters used. Throughout the training, the accuracy of the deep neural network reached 91.16%.

**Figure 14:**
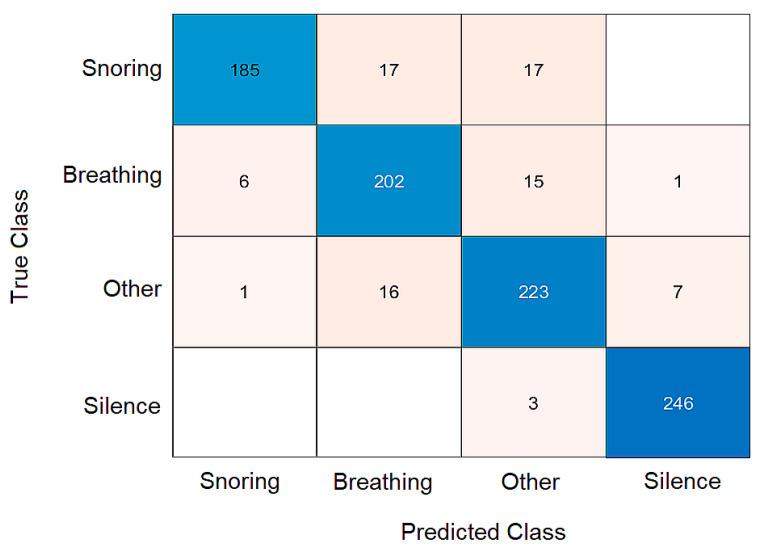
An obtained error matrix, vertical part: actual values, horizontal part: classifier output.

In Figure 14, there is an error matrix (confusion matrix) that shows the actual values of the predicted character in the rows and the classifier’s predictions in the columns. The cells of the matrix indicate the number of occurrences for each combination of actual and neural network predicted values. This error matrix was created using test data that was previously unknown to the neural network. For example, 246 sounds were correctly classified as quiet, and three sounds were incorrectly classified as others.

The parameters for the neural network (number of hidden units, learning rate, mini-batch size, number of epochs) were tested and adjusted during the model design and training process. Various architectures were also experimented with, such as an LSTM network with one LSTM layer and no dropout layers. Through experimentation, it was found that the neural network described above achieved the highest accuracy and is suitable for classifying sounds into different classes.

We used a proposed LSTM network for automatic classification. The classification process can be broken down into the following steps. The 60 min record is loaded and split into the first and second channels. Each 60 min record is further split in half to obtain four 30 min segments for smoother processing. The 30 min segments are divided into frames with a duration of one and a half seconds and an overlap of one second using the buffer function. Features are extracted from all frames, resulting in 3600 overlapping segments with 16,000 samples (sample rate = 16 kHz) for each 30 min segment for the entire hour and both channels; this results in four matrices of 16,000 × 3600. The neural network is loaded, and classification is performed using a for-loop. This loop gradually selects and classifies the second segments (frames with 16,000 samples) into their respective classes. The output is a categorical variable containing 3600 lines, with each line assigned to the appropriate category.

The number of occurrences of individual sound categories is based on physiology and empirical observations of the duration of snoring events, silence, and other sounds. The number of snoring sounds in a 30 min segment is obtained from the frequency matrix by searching all snoring segments. The characteristic sound of snoring usually appears in the inspiratory phase and lasts no longer than one second, followed by either silence or expiration. The algorithm for counting the occurrence of snoring events is optimized, and silence shorter than five seconds (after and before a snoring event) is excluded, as well as exhalation sounds (the exhalation immediately following snoring is not included in breathing sounds). This approach removes incorrectly classified and redundantly counted sounds from other categories. The number of breathing sounds is obtained similarly and optimized using if-conditions. The number of silences and other sounds is obtained similarly.

**Figure 15:**
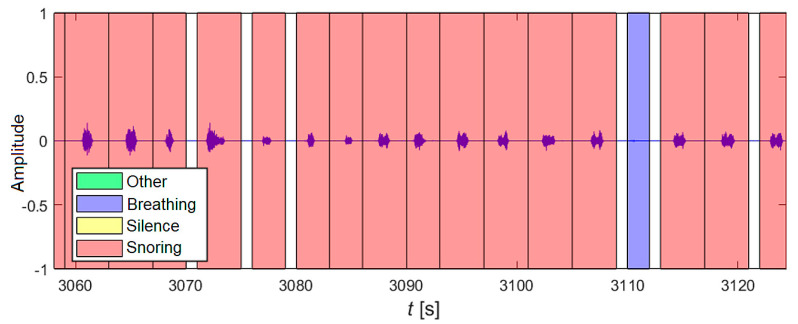
A selected section of the record after applying the classification using a neural network. Localized and classified events representing the sound manifestation of sleep are marked in colour.

The results can be visualized and analyzed. In this specific example, Figure 15 represents a selected time interval from the first hour of recording No. 3. During this time, snoring sounds (marked in red) and one breathing sound (marked in blue) were correctly classified.

## 5. Analysis of Results

To provide a more detailed assessment of the classifier’s performance, we evaluated additional metrics beyond overall classification accuracy (Table 4). Specifically, we calculated class-wise precision, recall, and F1-score for each of the four categories: snoring, breathing, silence, and other sounds. These metrics were derived from the confusion matrix presented in Figure 14, based on the validation dataset that was not used during training.

**Figure 16:**
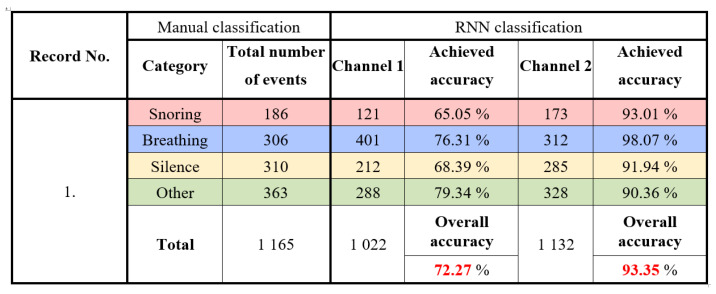
Experimental results: classification of the results for record No. 1.

Six all-night sound recordings were made during sleep at the Children’s Sleep Laboratory, UHM. Recordings numbered 1 to 5 were classified and analyzed. Record 6 was used for calibration, verification of the proposed method, and testing the optimization and parameters of the algorithms. All recordings were shortened to 5 h, and the sections at the beginning of the examination (calibration of PSG sensors) were removed. Each recording was manually scored by searching for sounds in the respective epochs, followed by neural network classification. The results obtained using manual scoring were compared with those using the designed classifier to determine the classifier’s accuracy for records 1 to 5. In Figure 16, the detailed classification results for record one are presented. A total of 1165 events were manually classified. The classification using RNN achieved an accuracy of 72.27% for channel 1 and 93.35% for channel 2.

**Figure 17:**
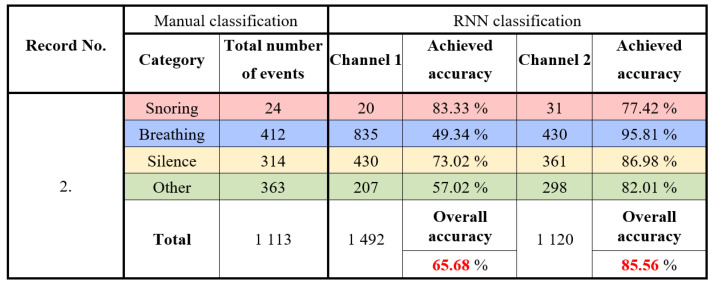
Experimental results: classification of the results for record No. 2.

In Figure 17 are the results for record No. 2. A parent was present during this all-night recording, which influenced the accuracy of the classification. A total of 1113 events were manually classified, and when the proposed classifier was applied, it achieved an accuracy of 65.68% for channel 1 and 86.56% for channel 2. For the third record, 5283 events were manually classified, and the proposed classifier achieved an accuracy of 73.31% for channel 1 and 92.19% for channel 2. The results for record No. 4 indicate that 2988 events were manually classified, with the classifier achieving an accuracy of 90.39% for channel 1 and 80.40% for channel 2. Lastly, the results for record No. 5, where 1759 sounds were classified using manual classification, and the classification using RNN achieved an accuracy of 79.96% for channel 1 and 92.16% for channel 2.

**Figure 18:**
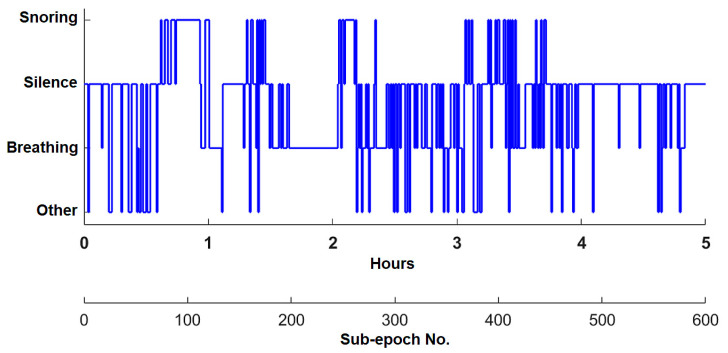
Experimental results: representation of sound occurrence as a function of time: SleepSoundGraph.

To visualize accurate sound classification of the recordings throughout the entire night, we can create a visual representation that shows the sound patterns during sleep. This representation of sound occurrence over time is entitled SleepSoundGraph, Figure 18. Each 30 s period, called a sub-epoch, contains specific sounds based on our classification. The dominant sound in each sub-epoch determines its category, allowing us to see the distribution of sounds throughout the night easily.

**Figure 19:**
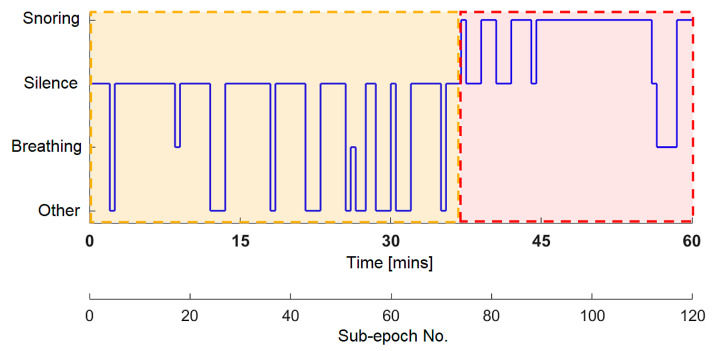
Experimental results: SleepSoundGraph of the first hour of recording No. 3.

Based on the graph in Figure 19 (one-hour excerpt from SleepSoundGraph Figure 18), we can see that during the first 35 min of this hour of sleep (highlighted in orange), there were primarily silent periods. However, starting from the 35th minute, the patient began snoring regularly (highlighted in red). These data provide an overview of the prevailing sounds during different minutes or sub-epochs of sleep.

**Figure 20:**
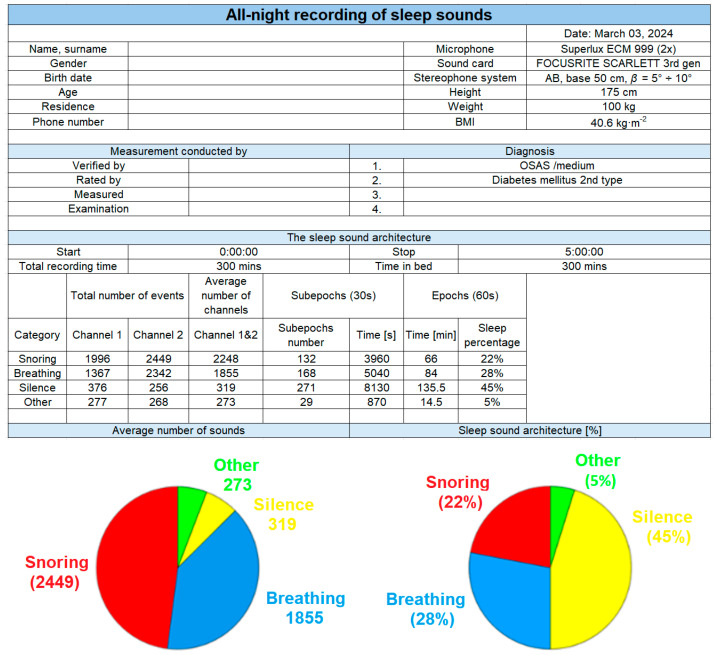
Experimental results: final output of the classification of all-night recording in the complete report.

The final output of the classification of the all-night recording is a report that includes basic information about the patient, the technique used, and the sound structure of sleep (Figure 20). This document is divided into segments in the form of tables. The first table displays basic information about the patient (name, surname, physiological parameters), the type of measuring microphone used, and the type of sound card. The subsequent table contains the names of healthcare professionals who may be involved in recording and evaluation. Adjacent to it, there is a box for recording the diagnoses for which the patient is being examined, or which are suspected. The report also contains: the start and end time, total recording duration, and the time the patient spends in bed. As this is a stereo recording, the total number of events recorded for each category (1: snoring, 2: breathing, 3: silence, and 4: other sounds) is documented for both channels and then averaged across channels. The number of sub-epochs indicates the prevailing sound in each 30 s segment. For example, if snoring is dominant in a particular sub-epoch, it is classified as a snoring sub-epoch. The Sleep Sound Architecture table includes a graph representing the percentage of various sub-epochs. For this recording, the breakdown is as follows: duration of the total sleep duration consisted of other sounds (speech, noise, door closing, etc.); time, which was dominated by sounds associated with silence, implying peaceful sleep with hushed breathing (intensity of approximately 20 dB); duration of sleep included sounds typical of breathing; the total recording duration which included snoring sounds. In the final section of the report, the SleepSoundGraph is plotted for the entire recording duration (5 h, 600 epochs), followed by graphs for each hour. From these graphic representations of the dependence of the occurrence of sounds of individual categories as a function of time, it is relatively easy to find out in which hour and in which sub-epoch, which sound dominated (where the given period belongs). In this way, clear information about the sound architecture of sleep is obtained. Based on the information in this report, it is possible to find an area of interest in the audio recording, replay it and analyze it further. If necessary, such a report can be supplemented with additional data (detailed analysis of each recording hour, graphical representations).

To assess the effectiveness of the proposed LSTM-based classification model, we compared it with two additional classifiers: a traditional machine learning model (SVM) and a deep learning model based on a CNN architecture. All models were trained and evaluated using the same dataset, comprising 1500 audio samples per class (6000 total), with identical training–validation splits (80:20). Feature extraction (MFCC, Mel-spectrogram, spectral centroid, and spectral slope) was applied uniformly across all models to ensure a relevant comparison. The results of the comparison, based on overall accuracy and macro-averaged F1-score, are summarized in Table 5.

## 6. Conclusions

Inadequate and poor-quality sleep can lead to chronic fatigue, lethargy, and excessive daytime sleepiness and can significantly impact social interactions and a person’s overall psychological state. Many individuals may not even realize that they are suffering from sleep disorders. This is why sleep medicine has become a highly discussed area in recent years. The classic all-night polysomnographic examination in sleep laboratories is demanding in terms of material, financial, and personnel resources. Therefore, researchers are working on developing a non-contact or partially contact polysomnographic methodology to reduce patient discomfort during this examination significantly.

In this study, a new method was developed. It offers an innovative approach to detecting, classifying, and quantifying sounds during all-night sleep recordings using stereophonic sound. The research was conducted on pediatric patients examined at the Children’s Sleep Laboratory of the Clinic for Children and Adolescents, Centre for Rare Sleep and Wake Disorders in Childhood, University Hospital Martin, Slovak Republic. This laboratory is the sole facility accredited by the Slovak Society of Sleep Medicine, a member of the Slovak Medical Association, and provides comprehensive diagnostic and therapeutic care for children with sleep disorders from infancy to 19 years of age throughout Slovakia.

The article discussed the design and implementation of a complete stereophonic all-night sound recording set-up. The system was first tested in an improvised laboratory and later calibrated for use in a specialized sleep lab. The pre-processing procedure included normalization, initial noise removal, and signal subsampling. In the signal processing phase, MATLAB was used to remove unwanted peaks and normalize the signal. Noise was further reduced through spectral subtraction methods. Subsequently, the recordings were manually reviewed, and the sounds were classified into four categories: snoring, breathing, silence, and other sounds. An RNN classification was employed, involving a neural network architecture with two layers of LSTM, which was trained using a dataset containing 1500 sounds from each category. The deep neural network achieved an accuracy of 91.16%. An algorithm for classification was developed, utilizing a trained neural network and optimization mechanisms to ensure the most accurate classification of sounds. The study’s output includes a report that provides a clear overview of the sleep sound architecture. The report also presents tables and graphical representations obtained from the sound recordings.

We observed significant variability in classification performance between channels. Channel 1 performed worse than channel 2 in some cases. This variability may be caused by various factors, such as differences in microphone placement, changes in their sensitivity, or different noise levels between channels. Future improvements to the system (such as advanced signal processing or data augmentation techniques) could help reduce these differences and thus improve the robustness of the system.

This study used a sample of six patients, limiting the statistical power and generalizability of our findings. The results demonstrated the effectiveness of our system. We recognize the need for a larger sample size for validation and broader application. We plan to expand the sample in the future to obtain more statistically significant results and improve the validity of our findings. However, in this work, our primary goal was to demonstrate the technical implementation and innovation of the system to help doctors assess sleep issues in people, with broader validation to be the subject of further research. The tool can also be used for at-home sleep monitoring, especially for patients with severe snoring.

## Figures and Tables

**Table 1:** Comparison of selected studies focused on sleep monitoring.

Study	Sensing Type	Age Group	Type	Objective	Accuracy
Cavusoglu et al. [4]	EEG	Adults	Contact	Sleep staging	85–90%
Dafna et al. [2]	Microphone	Adults	Contactless	Sleep staging	87%
Phan et al. [20]	DL on PSG data	Children	Contact	Sleep staging	88.8%
Our study	Stereo microphones	Children	Contactless	Sleep staging	91.16%

**Table 2:** Basic information about the subjects investigated.

Subject No.	Sex	Age [Years]	BMI [kg/m^2^]	Main Clinical Diagnosis
1	M	8	31.2	OSA (mild level)
2	M	5	17.2	OSA (mild level)
3	M	13	43.6	OSA (moderate level)
4	F	6	25.5	OSA (mild level)
5	M	14	41.3	OSA (mild level)
6	F	6	42.4	OSA (mild level)

**Table 3:** The number of sounds in the datasets and the division of the data into training and validation.

Category	Training (80%)	Validation (20%)	Dataset (100%)
Snoring	1200	300	1500
Breathing	1200	300	1500
Silence	1200	300	1500
Other sounds	1200	300	1500

**Table 4:** Per-class evaluation metrics for sleep sounds detection.

Class	Precision	Recall	F1 Score
Snoring	0.964	0.845	0.900
Breathing	0.860	0.902	0.880
Other sounds	0.864	0.903	0.883
Silence	0.969	0.988	0.978

**Table 5:** Comparative evaluation of classification accuracy and metrics across models.

Model	Accuracy	Macro Precision	Macro Recall	Macro F1 Score
Proposed Model	0.853	0.762	0.736	0.741
SVM	0.812	0.703	0.685	0.692
Random Forest	0.834	0.734	0.711	0.722
CNN	0.868	0.781	0.758	0.769

## Data Availability

The data that supports the findings of this study are available from the corresponding author (M. Smetana) upon reasonable request.

## Abbreviations

The following abbreviations are used in this manuscript:

AHI	Apnea–Hypopnea Index
ANN	Artificial Neural Network
BPTT	Back-Propagation Through Time
DAW	Digital Audio Workstation
DFT	Discrete Fourier Transform
EEG	ElectroEncephaloGraphy
ESN	Echo State Network
FIR	Finite Impulse Response
GDPR	General Data Protection Regulation
HMM	Hidden Markov Models
IFFT	Inverse Fast Fourier Transform
LSTM	Long Short-Term Memory
NREM	Non-Rapid Eye Movement
OSA	Obstructive Sleep Apnea
PSG	PolySomnoGraphy
REM	Rapid Eye Movement
RNN	Recurrent Neural Network
SNR	Signal-to-Noise Ratio
UHM	University Hospital Martin
WAV	Waveform Audio File Format
